# Delivery of Bioactive Compounds to Improve Skin Cell Responses on Microfabricated Electrospun Microenvironments

**DOI:** 10.3390/bioengineering8080105

**Published:** 2021-07-27

**Authors:** David H. Ramos-Rodriguez, Sheila MacNeil, Frederik Claeyssens, Ilida Ortega Asencio

**Affiliations:** 1 Bioengineering and Health Technologies Group, The School of Clinical Dentistry, University of Sheffield, Sheffield S10 2TA, UK; dhramosrodriguez1@sheffield.ac.uk; 2 Biomaterials and Tissue Engineering Group, Department of Materials Science and Engineering, Kroto Research Institute, University of Sheffield, Sheffield S3 7HQ, UK; s.macneil@sheffield.ac.uk (S.M.); f.claeyssens@sheffield.ac.uk (F.C.)

**Keywords:** topography, electrospinning, 2-deoxy-D-ribose, 17β-estradiol, aloe vera

## Abstract

The introduction of microtopographies within biomaterial devices is a promising approach that allows one to replicate to a degree the complex native environment in which human cells reside. Previously, our group showed that by combining electrospun fibers and additive manufacturing it is possible to replicate to an extent the stem cell microenvironment (rete ridges) located between the epidermal and dermal layers. Our group has also explored the use of novel proangiogenic compounds to improve the vascularization of skin constructs. Here, we combine our previous approaches to fabricate innovative polycaprolactone fibrous microtopographical scaffolds loaded with bioactive compounds (2-deoxy-D-ribose, 17β-estradiol, and aloe vera). Metabolic activity assay showed that microstructured scaffolds can be used to deliver bioactive agents and that the chemical relation between the working compound and the electrospinning solution is critical to replicate as much as possible the targeted morphologies. We also reported that human skin cell lines have a dose-dependent response to the bioactive compounds and that their inclusion has the potential to improve cell activity, induce blood vessel formation and alter the expression of relevant epithelial markers (collagen IV and integrin β1). In summary, we have developed fibrous matrixes containing synthetic rete-ridge-like structures that can deliver key bioactive compounds that can enhance skin regeneration and ultimately aid in the development of a complex wound healing device.

## 1. Introduction

The regeneration of human skin is vital for its correct functioning as the first protective barrier against the external environment. Skin injuries can be caused by constant wear, exposure to chemical contamination and heat or high energy electromagnetic radiation among others. Skin injuries are normally categorized in terms of the damage caused to the skin structure (superficial, partial-thickness, or full-thickness) [1,2]. According to their category, these injuries are normally treated using wound excision and skin grafting (for mild to severe injuries) or by the use of skin substitutes and wound dressings for superficial and partial thickness injuries [3,4].

The ideal wound dressing aids skin healing by restoring barrier function. The dressing should have proper adhesion to the wound site, offer a 3D environment that allows cell-cell and cell-extracellular matrix (ECM) interactions, enable cellular infiltration and encourage vascularization [5,6,7,8]. Vascularization is critical to provide nutrients and oxygen, to avoid infection or rejection and to preserve tissue function. The formation of new vasculature (angiogenesis) plays a key role in the wound healing process [9,10], however, the slow growth rate of neovasculature (approximately 5 µm/h) is normally insufficient for large tissue constructs [11]. Therefore, introducing angiogenic agents into skin wound dressings has the potential to improve tissue regeneration when the microvasculature on the skin is severely compromised.

A powerful approach for the introduction of biological agents within scaffolds for skin tissue regeneration is the use of electrospinning [5,12,13,14]. Electrospinning is a versatile advanced manufacturing technique that allows the creation of high-surface fibrous membranes that can also be used as drug delivery carriers [15,16,17]. The combination of manufacturing techniques such as microstereolithography and electrospinning has also been explored to increase construct complexity by creating micropatterned fibrous membranes [18,19]. These membranes have been used to recreate the microarchitecture of the stem cell microenvironment including cornea [18] and bone [20] among other tissues. Specifically for skin, the fabrication of constructs containing artificial microenvironments that mimic the dermal-epidermal junction (DEJ) has recently attracted special attention due to the potential to improve skin regeneration shown by this approach [21,22]. The 3D microstructure of the DEJ consists of dermal papillae protrusions called rete ridges that have mechanical and biological functions in the human skin [23,24,25].

The rete ridges create stem cell microenvironments that protect the epidermal stem cell population that resides within and govern cell behavior by cell-cell, cytokine-cell, or cell-ECM interactions [26,27]. The stem cell microenvironment uses structural (micropockets), biological (integrin and non-integrin receptors), and chemical (soluble factors) cues to protect and regulate populations of adult stem cells [28,29,30,31,32] that dictate tissue homeostatic balance and regeneration [33,34,35,36]

Our group has recently reported that polycaprolactone (PCL) electrospun fibers can be combined with 3D printed collectors to produce fibrous artificial microenvironments that improve cell proliferation of human dermal keratinocytes, induce the production of Iβ1 and can recreate to a degree the structures that mimic the rete ridges on a skin tissue engineering model [21]. 

In this work, we have used our previously developed rete-ridge-like microfabricated scaffolds with a focus on the capabilities of electrospun membranes to deliver angiogenic agents. We have selected three relevant bioactive compounds that have been studied before for their potential as angiogenic agents: 2-deoxy-D-ribose (2dDr), estradiol (E2), and aloe vera (AV). 2dDr and E2 have been previously used by our group as potent proangiogenic agents capable of inducing vascularization without some of the drawbacks of the current gold standard VEGF (vascular endothelial growth factor) such as short half-life and high fabrication cost [37,38,39]. Moreover, AV has been previously included within electrospun membranes [40], its regenerative, anti-inflammatory, and angiogenic effects [41] make it a promising compound to be used for skin regeneration applications.

The aim of our work was to evaluate the feasibility of including angiogenic agents within microfabricated rete-ridge-like electrospun scaffolds. For this, we optimized the manufacturing route to ensure the addition of the bioactive compounds as well as retention of the desired rete-ridge-like morphology. Moreover, our constructs were tested in vitro using relevant skin cell lines (epidermal keratinocytes and dermal fibroblasts) and we also evaluated their angiogenic potential using the ex-ovo chick chorioallantoic membrane (CAM) assay.

## 2. Materials and Methods

### 2.1. Polymer Solution Preparation

Polymer solutions of PCL (Mn = 80,000 g/mol) were prepared prior to the electrospinning process. Solutions were made using a solvent system composed of dichloromethane (DCM) and dimethylformaldehyde (DMF). 12% PCL weight/weight (*w*/*w*) solutions were prepared by dissolving 1 g of polymer with their solvent system as specified. In the case of AV, dimethyl sulfoxide (DMSO) was used to solubilize the compound. Table 1 shows the polymer solutions prepared for the electrospinning process. Solutions were cover in parafilm and left in a see-saw rocker (SSL4, Stuart™, Staffordshire, UK) for 24 h at room temperature (RT) to homogenize the polymer solution.

### 2.2. Electrospinning

A vertical electrospinning set-up was used to fabricate both random electrospun scaffolds (RES) and topographically controlled electrospun scaffolds (TCES).

#### 2.2.1. Fabrication of Random Electrospun Membranes (RES)

Fabrication of RES was carried out by using 1 mL syringes with 20 gauge 1″ syringe tips (8001213, Fisnar, Germantown, WI, USA) loaded with ~1.1 mL of the polymer solution. The syringe was loaded into a syringe pump (PHD 22/2000, Harvard Apparatus, Holliston, MA, USA) and the syringe cap was connected to a voltage supply. The static collector was covered with silicon-coated paper and placed at a distance of 12 cm from the needle tip. Flow rate and voltage are detailed for every solution using their code as a reference in Table 2. Electrospun membranes were stored at 4 °C in sealable bags.

#### 2.2.2. Fabrication of Topographically Controlled Electrospun Scaffolds (TCES)

**Figure 1:**
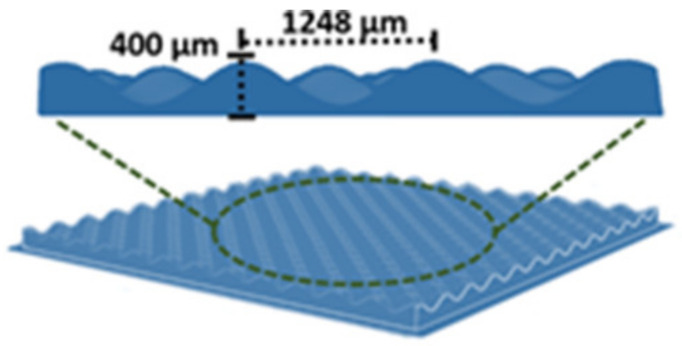
The patterned collector used to fabricate TCES loaded with bioactive compounds.

As we have described previously [18,21], the fabrication of TCES was carried out by combining 3D printed pattern collectors and conventional electrospinning. To examine the effects of loading bioactive compounds, a single patterned collector was used to fabricate all scaffolds. Figure 1 shows the dimensions of the patterned collector. Table 3 shows the flow rate and voltage used to fabricate TCES for each polymer solution. Approximately 200 µL of the polymer solution were electrospun on top for every collector. The resulted TCESs were cut before being peeled from the patterned collector to avoid structural damage to the scaffold.

### 2.3. Characterization of Electrospun Membranes

#### 2.3.1. Scanning Electron Microscopy (SEM)

TCES and RES scaffolds were coated with a 10 nm gold layer (SC 500A, Emscope, Heathfield, UK) and mounted on aluminum pin stubs (AGG301, agar scientific) with carbon tabs (AGG3347N, Agar Scientific, Stansted, UK). Scaffolds were analyzed by scanning electron microscopy (SEM) FE SEM, JSM-6500F (JEOL, Tokyo, Japan) and FE/VP SEM, TM3030Plus (Hitachi, Tokyo, Japan ). Spot size and voltage were set up at 10 kV and 3.5 nm respectively for all samples. ImageJ software v. 1.48 from the National Institutes of Health (NIH, Bethesda, MD, USA) [42] was used to measure fiber diameter on the SEM micrographs. Three independent tests were performed for each material with 15 fibers measured for each micrograph (N = 3, n = 15).

#### 2.3.2. Uniaxial Tensile Testing

Mechanical testing on RES was performed using a motorized force tester MultiTest-dV (Mecmesin, Slinfold, UK). A 25 N load cell (ELS 25 N, Mecmesin) was used to perform a uniaxial tensile test. Vector Pro software (version 6.2.0, Mecmesin) was used to control the force tester and for data analysis. Samples were cut as square pieces of 8 mm width by 22 mm length to use 5 mm of each side to grip the samples and allowed a 12 mm gap between the grips. The test speed was set up as 6 mm/min and the thickness of the samples was measured to normalize the data. Maximum stress versus strain were recorded for each material. Two independent tests were performed by triplicate for each material (N = 2, n = 3).

### 2.4. Differential Scanning Calorimetry and Contact Angle Analysis

The thermal performance of RES was evaluated by differential scanning calorimetry (DSC). Thermograms of RES (N = 2, n = 3) were obtained using a PerkinElmer DSC 4000 (PerkinElmer, Waltham, MA, USA). Nitrogen gas flow was of 20 mL/min and a scanning rate of 10 °C from 30 °C to 100 °C (holding 30 °C for 1 min). The weight of all the samples was recorded before the DSC analysis. Additionally, to observe any changes in wettability due to the incorporation of bioactive compounds, A drop shape analyzer DSA100E (KRÜSS, Hamburg, Germany) was used to calculate the water contact angle. RES were cut as 8 by 8 mm square pieces and a 5 µL droplet of distilled water was placed on top. A digital camera and KRÜSS software were used to capture and process the data. Two independent tests were performed by triplicate for each material (N = 2, n = 3).

### 2.5. In Vitro Cell Culture—Bioactive Compound in Solution

Skin human primary cell lines were used to study the effects in vitro of the 2dDr, E2, or AV. Human dermal fibroblasts (HDF) and human epidermal keratinocytes (HDK) were isolated from skin as described by Gosh et al. [43]. The skin used was obtained from patients undergoing elective breast reductions and abdominoplasties who gave informed consent for use of their excised skin for research purposes through the Sheffield hospital directorate of Plastic, Reconstructive Hand and Burns surgery research ethics number 15/YH/0177 under the Human Tissue Authority 12179.

Metabolic activity and cell proliferation were evaluated for HDF and HDF + HDK when culture with 2dDr, E2, or AV in solution. For the monoculture of HDF, 3000 cells were seeded per well, whereas for HDF + HDK a ratio of 1:3 was followed with 750 HDF + 2250 HDK seeded per well. Cells were seeded in 48-well plates using DMEM for HDF and Green’s medium for HDF + HDK (see Supplementary Table S1 for all cell medium supplements). Different concentrations of each bioactive compound were dissolved on their respective media. The concentrations used were 50, 100, 500, and 1000 µM for 2dDr; 10, 50, 100, and 200 µM for E2; and 1, 5, 10, and 20 mg/mL for AV. Because of E2 low solubility in water, DMSO was used to create the media solutions and a control was placed to compare any changes in cell behavior. A resazurin reduction assay and DNA quantification Picogreen™ (P758, Fisher, Waltham, MA, USA) assay were performed on days 1, 4, and 6.

On the day of the experiment, a 10% resazurin solution in cell media was prepared from a 1 mM in PBS resazurin stock (R7017) and incubated for 4 h before reading fluorescence (λ_ex_ = 540 nm, λ_em_ = 630 nm) using a FLx800 spectrophotometer (Bio-Tek Instruments Inc., Winooski, VT, USA). The Picogreen™ assay was performed immediately after the resazurin assay. The resazurin solution was removed and a 1× Tris-EDTA buffer was used to induce cell lysis using the freeze–thaw method. Once completely defrosted, 250 µL of the Picogreen™ working solution were added and the fluorescence signal for each sample was read at λ_ex_ = 480 nm and λ_em_ = 520 nm using the FLx800 spectrophotometer plate reader. Three independent tests were performed by triplicate for each experiment (N = 3, n = 3).

### 2.6. Air Plasma Treatment for TCES and RES

Air plasma surface treatment was performed to enhance cell adhesion on both TCES and RES. Prior to cell culture, the scaffolds were cut into 15 × 15 mm square pieces and placed into a Zepto plasma cleaner (Diener Electronic, Ebhausen, Germany) set with low-power parameters of 10 W–40 KHz and exposure time of 2 min. TCES and RES were stored at 4 °C in sealable bags to avoid any degradation of the plasma coating.

### 2.7. In Vitro Cell Culture—Bioactive Compound in Loaded in TCES and RES

Metabolic activity of HDF on RES, and HDF + HDK seeded on TCES was evaluated using the resazurin reduction assay described before on days 1, 4, and 6. On the day of cell culture, a metal ring was placed on top of each scaffold to hold 500 µL of a cell suspension containing 300,000 HDK + 100,000 HDF for TCES and 100,000 HDF for RES. Samples were cultured at 37 °C in a 5% CO_2_ humidified atmosphere. After 24 h, the metal ring was removed, and the scaffolds were transferred to a new 6-well plate to perform the metabolic assay. Two independent tests were performed by triplicate per experiment (N = 2, n = 3). TCES and RES were fixed at day 6 using 3.7% paraformaldehyde solution incubated for 1 h. Samples were stored in PBS at 4 °C for further analysis.

### 2.8. Immnolabelling and Cell Imaging

Sample preparation for lightsheet microscopy (Lightsheet Z.1, Carl Zeiss, Baden-Württemberg, Germany) was performed as described in our previous work [21]. RES and TCES seeded with HDF + HDK were stained for cell nuclei using 4′,6-diamidino-2-phenylindole (DAPI) and immunostained for COL IV (ab6586, abcam, Cambridge, UK) and integrin β1 (Iβ1) (ab24693, abcam). Anti-rabbit Alexa Fluor^®^ 488 (ab150077, abcam) and anti-mouse Alexa Fluor^®^ 647 (ab150115, abcam) secondary antibodies were incubated for COL IV and Iβ1 respectively at concentrations of 1:500 in 1% BSA for 4 h at room temperature.

TCES and RES were mounted into a 2.15 mm glass capillary using a 1% *w/v* agarose solution (A9414, Sigma, St Louis, MO, USA). A Zeiss W Plan-Apochromat 10×/NA 0.5 lens was used for detection and a Zeiss LSFM 5×/NA 0.1 lens for illumination. Lasers 638 nm, 488 nm, and 405 nm were used for excitation of Alexa Fluor^®^ 647, Alexa Fluor^®^ 488, and DAPI respectively. For emission, band pass 545–590, low pass 660, and short band 550 filters were used. The ZEN 3.0 SR (black) (version 16.0.2.306) software (2012, Carl Zeiss) was used for image acquisition and processing.

### 2.9. Chick Chorioallantoic Membrane (CAM) Assay

The performance of RES as carriers of 2dDr, E2, and AV was studied using the ex-ovo chick chorioallantoic membrane (CAM) assay as described by Mangir et al. [44]. Pathogen-free fertilized white leghorn chicken eggs (*Gallus gallus domesticus*) obtained from Henry Stewart Co. Ltd. (Fakenham, UK) were incubated and handled under the guidelines of the Home Office, UK.

On embryonic development day (EDD) 0, eggs were incubated in a humidified hatching incubator (Rcom King Suro Max-20, P&T Poultry, Wichita, KS, USA) at 38 °C. Cracking of the eggshells on square weigh boats was executed on EDD 3. The weighting boats were placed inside sterile petri dishes and filled with 3 mL of PBS + 1% penicillin–streptomycin solution (100 IU/mL–100 mg/Ml). The eggs were cracked inside the weighting boats and placed immediately after inside a humidified incubator (Binder, Tuttlingen, Germany) at 38 °C. The embryos were left inside the incubator for the rest of the experiment (from EDD 3 to EDD 12). Embryos were checked daily for any malformation or infection. Electrospun scaffolds were implanted on EDD 7. For implantation, 8 mm disks were cut from the electrospun mats and implanted within the boundaries of the CAM. Two independent tests were performed with five samples per experiment (N = 2, n = 5).

Images were captured on EDD 12 using a digital camera and MicroCapture software (version 2.0). To increase the contrast between the blood vessels and the sample, a white hydrating body cream (Neutrogena, Notthingham, UK) was injected into the surrounding area of the sample. After imaging, embryos were sacrificed by either decapitation or bleeding. ImageJ software v. 1.52 [42] was used to process and analyze blood vessel formation. The image was split in intro its RBG components, preserving the green channel for “Mexican Hat” filtering, contrast-enhancing, and unmask filtering. The resulting image was then transformed to binary and processed using the vessel analysis tool [45] to quantify vascular density.

### 2.10. Statistical Analysis

GraphPad Prism software (version 9.10, San Diego, CA, USA) was used to performed statistical analyses using one-way analysis of variance (ANOVA) following by Tukey’s multiple comparisons tests. In all cases, *p* values < 0.05 were considered as statistically significant.

## 3. Results

### 3.1. Characterization of RES Physiochemical Properties

**Figure 2:**
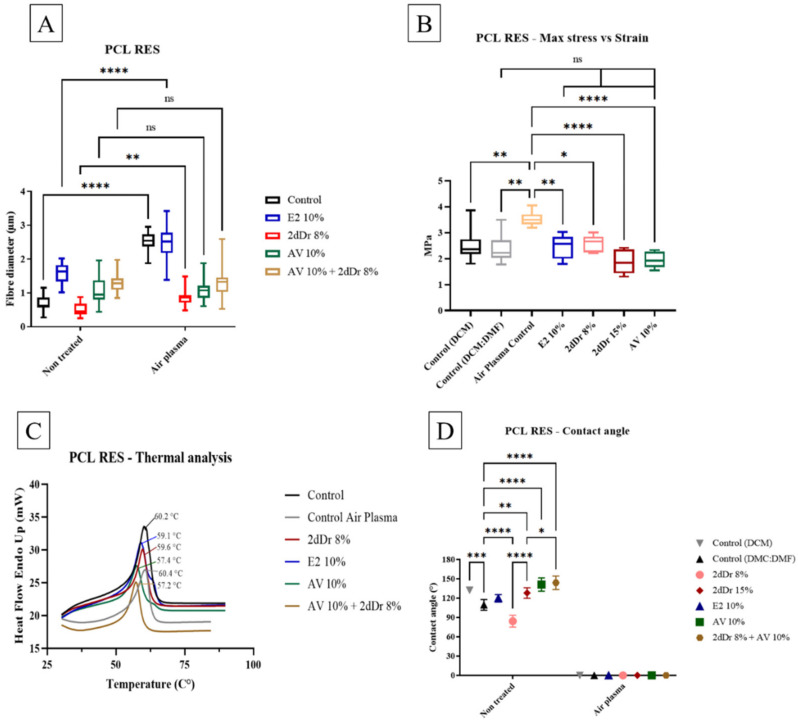
Physicochemical characterization of PCL RES. (**A**) Fiber diameter of PCL RES before and after air plasma treatment (N = 3, n = 15). (**B**) Tensile strength of PCL RES controls and loaded with bioactive compounds. (**C**) DCS of PCL RES loaded with bioactive compounds. (**D**) Contact angle of PCL RES loaded with bioactive compounds, before and after air plasma treatment. * *p* < 0.05, ** *p* < 0.01, *** *p* < 0.001, **** *p* < 0.0001. N = 2, n = 3.

Fiber diameter, tensile strength, DSC, and wettability characterization were performed for PCL RES. The fiber diameter of PCL RES and air plasma treated PCL RES was measured E2, 2dDr, and AV-loaded scaffolds. Fiber diameter increased significantly in PCL RES that contained E2 and 2dDr after air plasma treatment. On the other hand, the fiber diameter of PCL RES loaded with AV was not significantly different after air plasma treatment (Figure 2A). Furthermore, tensile strength of air plasma-treated PCL RES was significantly higher in comparison to other test groups (Figure 2B). Interestingly, the tensile strength of PCL RES control (DCM:DMF) was not significantly different in comparison to PCL RES loaded with bioactive compounds. Regarding DCS, the melting point of PCL RES ranged between 57.2 °C and 60.4 °C. PCL RES loaded with AV + 2dDr registered the lowest melting point, whereas PCL RES air plasma control had the highest melting point (Figure 2C). The wettability of PCL RES was different depending on which bioactive compound was loaded. PCL RES loaded with 2dDr 8% has the lowest contact angle when compared to other PCL RES. In addition, contact angle PCL RES control (DCM:DMF) was significantly different in comparison to other groups, except to PCL RES E2. However, after PCL RES were air plasma treated, the contact angle of all groups was 0° (Figure 2D).

### 3.2. Characterization of TCES Loaded with Bioactive Compounds

**Figure 3:**
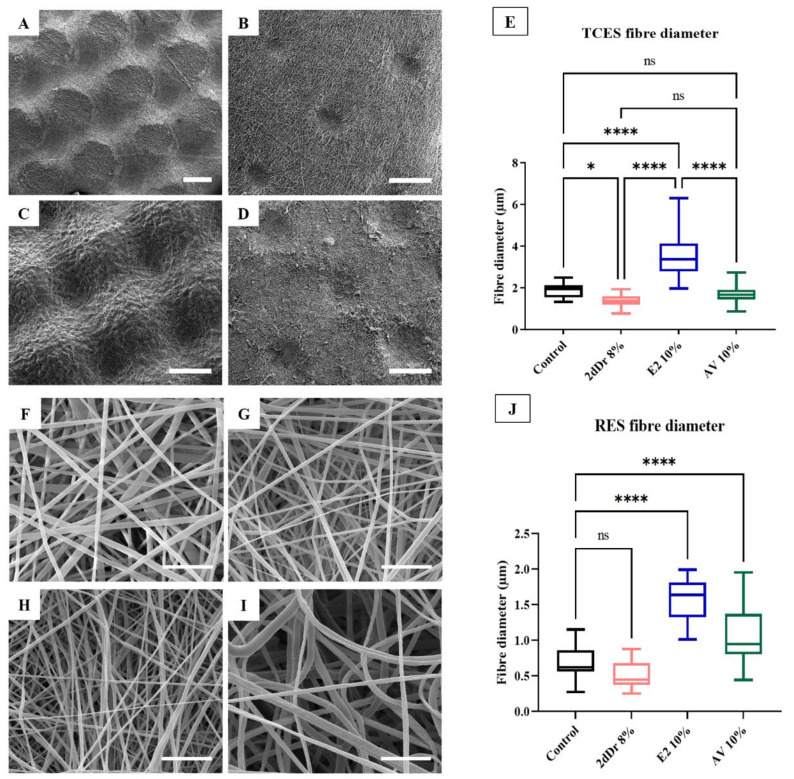
SEM micrographs of PCL TCES fabricated using the same patterned collector. (**A**) Control, (**B**) 8% 2dDr, (**C**) 10% E2, and (**D**) 10% AV. (**E**) Box plot of the TCES fiber diameter. SEM micrographs of PCL RES. (**F**) Control, (**G**) 8% 2dDr, (**H**) 10% E2, and (**I**) 10% AV. (**J**) Box plot of the RES fiber diameter. ns = no significance, * *p* < 0.05, **** *p* < 0.0001. N = 3, n = 15.

Fabrication of TCES loaded with bioactive compounds is shown in Figure 3. SEM micrographs of TCES fabricated with the same patterned collector showed the effects of introducing bioactive compounds on the capacity of the electrospun fibers to mimic the microstructure of the collector. Compared to the pure TCES PCL control, only the TCES loaded with 10% wt. E2 were able to preserve the ridged morphology of the collector, with AV 10% wt. and 2dDr 8% wt. showing only small features that resemble the tip of the collectors. Average fiber diameter was about 1.5 µm for all TCES except for the scaffolds loaded with 10% wt. E2 showed an increase in average fiber diameter up to 3.5 µm. Furthermore, all scaffolds showed smaller fiber diameters when electrospun as RES with the E2 showing a similar trend of increase in fiber diameter as with the TCES.

### 3.3. In Vitro Cell Response of HDF and HDK to Solubilized Bioactive Compounds

**Figure 4:**
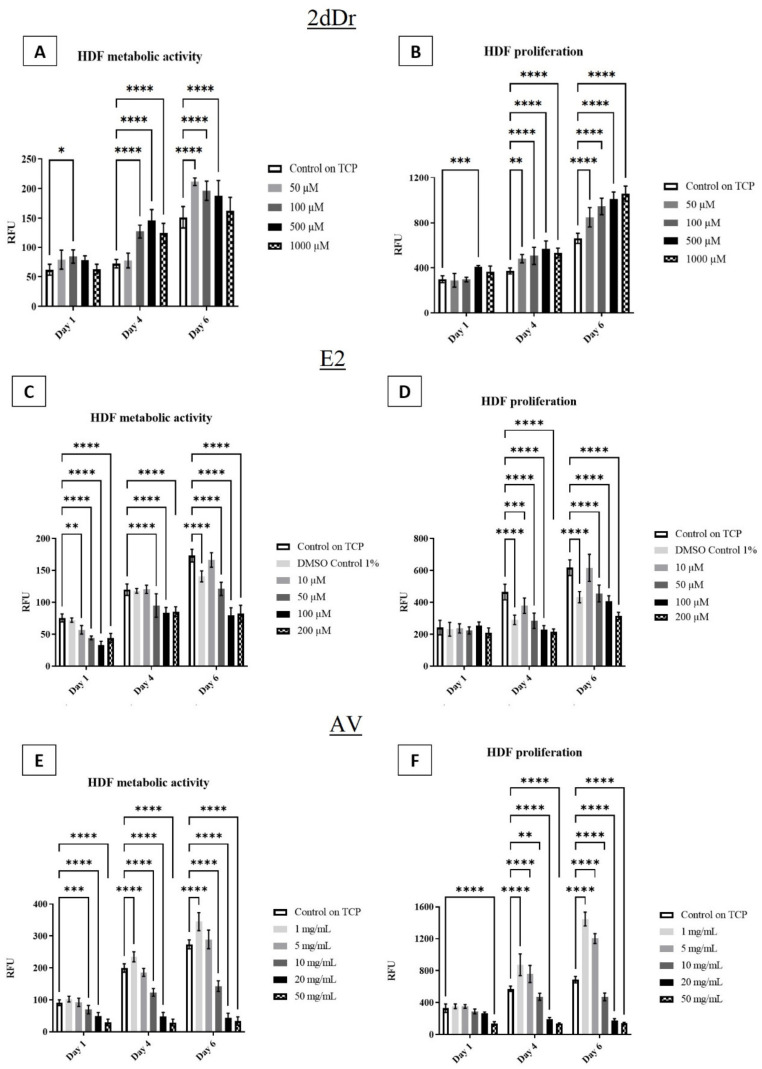
HDF metabolic activity and proliferation in the presence of solubilized 2dDr (**A**,**B**), E2 (**C**,**D**), and AV (**E**,**F**) at different concentrations. * *p* < 0.05, ** *p* < 0.01, *** *p* < 0.001, **** *p* < 0.0001. N = 3, n = 3.

**Figure 5:**
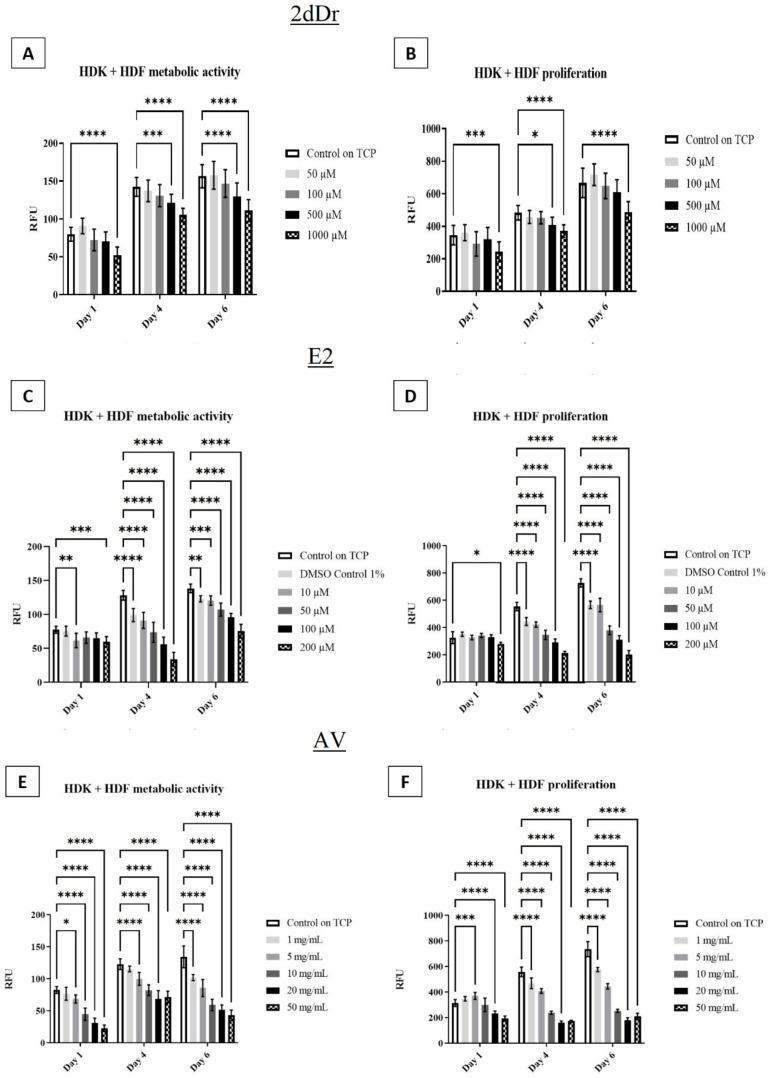
HDK + HDF metabolic activity and proliferation in the presence of solubilized 2dDr (**A**,**B**), E2 (**C**,**D**), and AV (**E**,**F**) at different concentrations. * *p* < 0.05, ** *p* < 0.01, *** *p* < 0.001, **** *p* < 0.0001. N = 3, n = 3.

The effects of the bioactive compounds on cell metabolic activity and proliferation were evaluated by exposing HDF to different concentrations of the solubilized bioactive compounds. Figure 4 shows that low concentrations of both 2dDr and AV have a positive effect on HDF cell proliferation and metabolic activity when compared to the control group. However, E2 did not show any significant increase in metabolic activity or proliferation when used in low concentrations. In comparison to E2 and AV, 2dDr showed an increase in cell proliferation and metabolic activity at all tested concentrations, while higher concentrations of AV or E2 reduced HDF cell activity. The co-culture of HDK + HDF showed similar behavior in the presence of solubilized E2 but not an increase in either metabolic activity or proliferation when exposed to 2dDr or AV as observed for HDF (Figure 5).

### 3.4. In Vitro Cell Culture of RES and TCES Loaded with Bioactive Compounds

**Figure 6:**
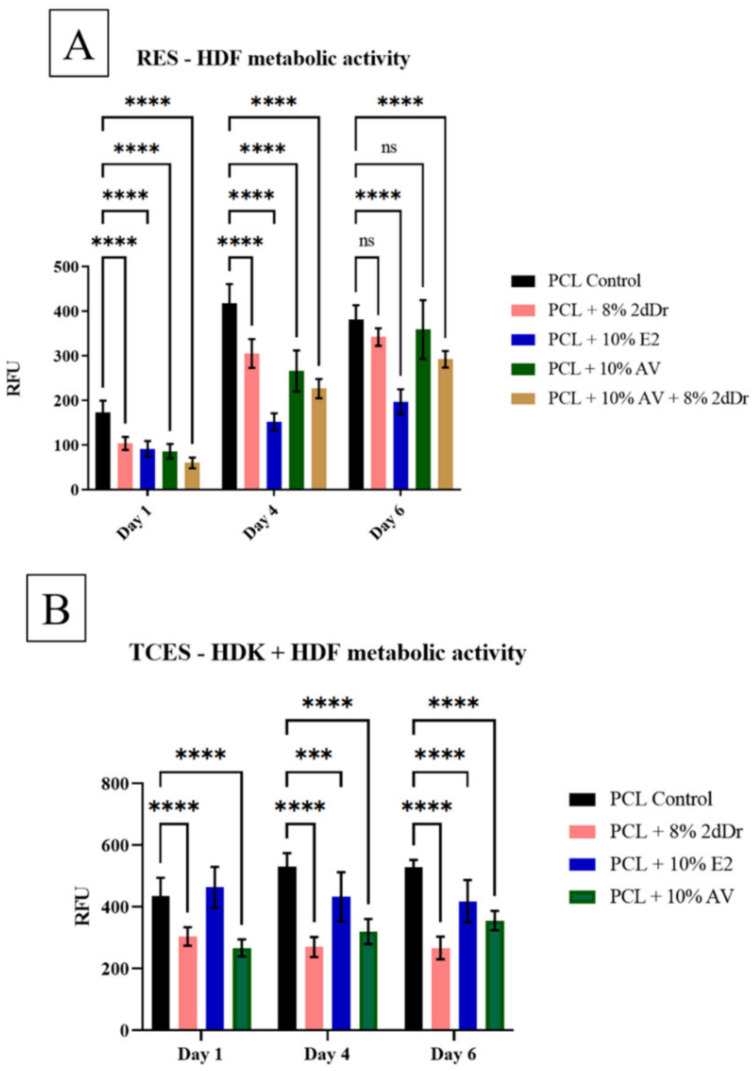
Cell metabolic activity of (**A**) HDF seeded on PCL RES loaded with different bioactive compounds and (**B**) HDF + HDK seeded on PCL TCES loaded with 2dDr, E2, or AV. *** *p* < 0.001, **** *p* < 0.0001. N = 2, n = 3.

The effects of electrospun scaffolds loaded with bioactive compounds were evaluated by measuring the metabolic activity of HDF when seeded in RES and HDK + HDF when seeded on TCES. Figure 6 shows that RES scaffolds loaded with 8% 2dDr and 10% AV did not cause any significant changes in HDF metabolic activity by day 6. In contrast, cells seeded on RES loaded with 10% E2 and 10% AV + 8% 2dDr showed a decrease in metabolic activity compared to the control group. Furthermore, all groups of HDK + HDF seeded on TCES loaded with bioactive compounds showed a decrease in metabolic activity when compared with the control group.

**Figure 7:**
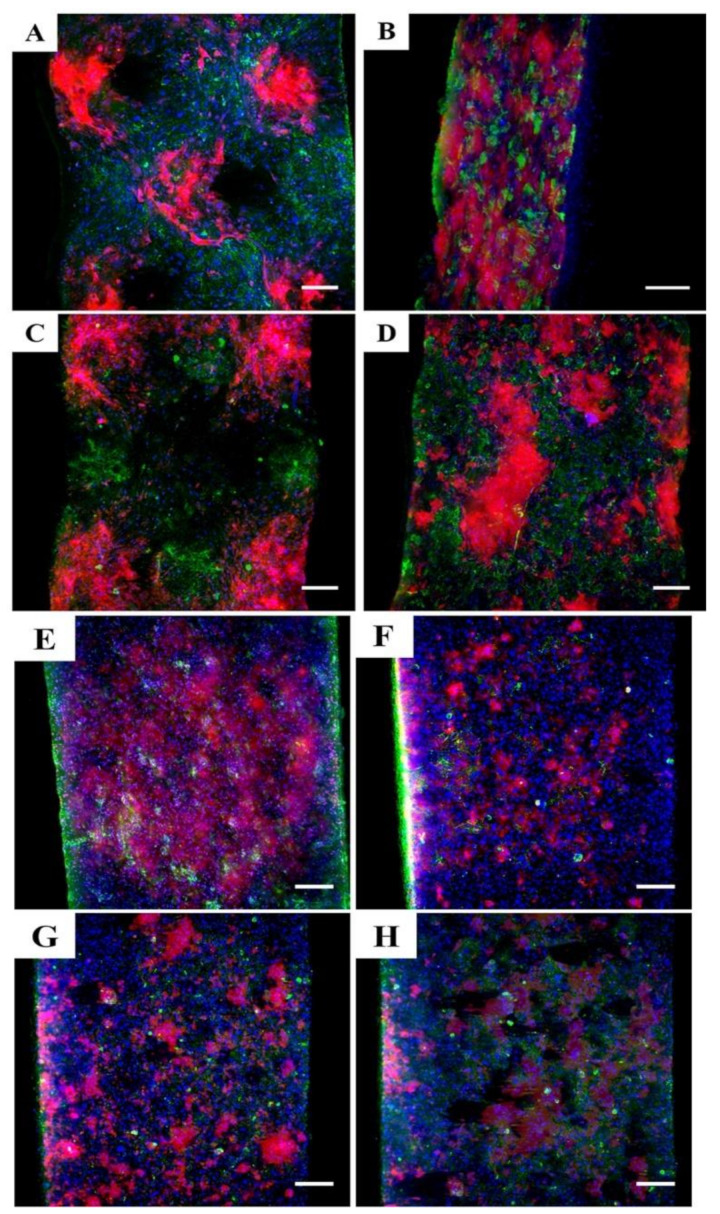
Lightsheet microscopy images of TCES seeded with HDF + HDK and fabricated with (**A**) PCL control, (**B**) 8% 2dDr, (**C**) 10% E2, and (**D**) 10% AV. RES seeded with HDF + HDK and fabricated with (**E**) PCL control, (**F**) 8% 2dDr, (**G**) 10% E2, and (**H**) 10% AV are also shown. Samples were immunostained for Iβ1 (red), COL IV (green), and DAPI (blue). Scale bar = 200 µm.

Figure 7 shows Lightsheet microscopy images of HDK + HDF seeded on TCES at day 6. The immunostaining of cell nuclei, Iβ1, and COLIV shows that clusters of dermal cells are well defined on the scaffolds that better preserve the microstructure of the collector. Moreover, TCES loaded with 2dDr and AV showed expression of Iβ1 and proliferation of cells without any distinctive pattern when compared with the E2 and control groups.

### 3.5. Evaluation of Angiogenic Potential by CAM Assay

**Figure 8:**
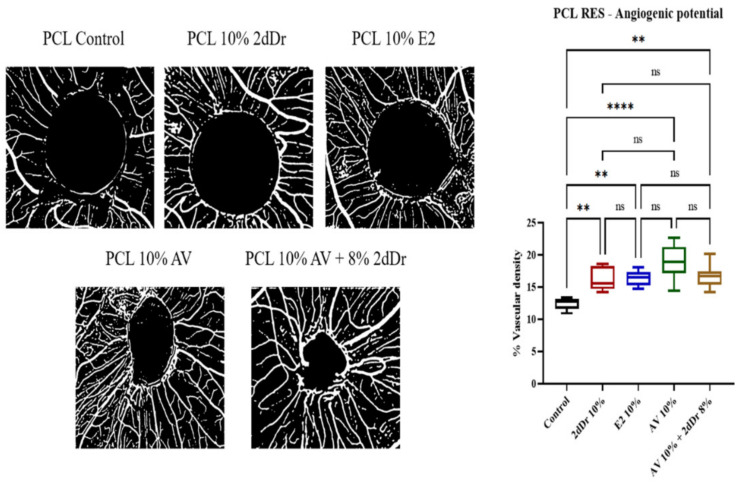
Evaluation of angiogenic potential for PCL scaffolds loaded with bioactive compounds. Negative images of the vessel formation (left) and the % vascular density (right) for each bioactive compound and combination tested are shown. ns = no significance, ** *p* < 0.01, **** *p* < 0.0001. N = 2, n = 5.

The ability of the RES scaffolds to produce an angiogenic response was evaluated by analyzing the vascular density around the sample implanted on the CAM. Figure 8 shows that RES loaded with 8% wt. 2dDr, 10% wt. E2 and 10% wt. AV showed a significant increase in vessel formation in comparison with the control group. PCL scaffolds loaded with 10% wt. AV showed the highest angiogenic response compared to the control.

## 4. Discussion

The fabrication of basic PCL microfabricated scaffolds for mimicking aspects of the rete ridge environments has been recently described by our group [22]. In this study, we present different strategies to develop microfabricated scaffolds with increased biofunctionality via using the delivery of angiogenic agents 2dDr, E2, or AV. The inclusion of biofunctional agents within the electrospinning process required careful optimization of the parameters and in some cases, the desired microstructure cannot be achieved without significantly changing the polymer solution properties [16,46,47]. To fabricate the TCES it is critical to control the directionality of the electrospinning jet to force the electrospun fibers to populate the microfeatures of the collector. Although the fabrication of electrospun scaffolds to deliver bioactive compounds has been explored and tested before by several authors [16,48,49], the need for a stable and directed jet to fabricate TCES requires further optimization of the electrospinning parameters.

Concentrations higher than the ones used here (8% wt. 2dDr, 10% wt. E2, or 10% wt. AV) failed to produce microtopographical cues on the scaffolds due to the formation of non-beaded fibers and aggregates that limited the production of microfeatures. This behavior can be related to disturbances of the electrohydrodynamic forces (surface tension, viscosity, and electrostatic repulsion) that interact when the Taylor cone is being formed [17].

Previously, we studied the differences in electrospinability and cell behavior across several topographies. However, we have now focused our efforts on using a single micropatterned collector to fabricate bioactive microfabricated scaffolds that include angiogenic agents [21]. Our results showed that solutions loaded with the non-polar hydrophobic E2 produced scaffolds with well-defined microfeatures. This behavior is related to the chemical composition of the bioactive compound and thus its solubility with the solvent DCM:DMF. Because E2 is completely solubilized within the electrospinning solution the electrospun fibers were able to completely populate the patterned collector as the PCL control. In contrast, the polar hydrophilic 2dDr induced a more spread fiber deposition that resulted in less defined microfeatures. Because AV is a complex mixture of polar and non-polar compounds [50], the fabricated microfeatures within the fibrous scaffolds were more defined than those produced by 2dDr but less efficient than E2.

There were no significant changes in mechanical or thermal properties among the PCL scaffolds loaded with bioactive compounds as shown by the tensile testing and DSC analysis, respectively. The slight change in T_m_ indicates that the bioactive compounds are causing a change in the semi-crystalline structure of the PCL as has been reported before [51]. Regarding fiber diameter, TCES showed higher values than their RES counterpart. This change in diameter is primarily related to the increase in flow rate needed to fabricate the TCES [21,48]. However, upon plasma treatment, the scaffolds showed a change not only in tensile strength but in fiber diameter and most importantly in hydrophobicity (when water contact angle was measured). Because hydrophilic scaffolds allow for rapid cell attachment this aspect is critical for most tissue engineering applications [52].

Our data showed that although small changes in surface contact angle are possible by loading the bioactive compounds on the electrospun scaffolds, plasma treatment of hydrophobic polymers such as PCL is critical to achieve rapid cell attachment. However, as it happens with the mechanical properties, the differences in contact angle between RES and TCES would need to be fully characterized to determine any differences caused by the fabrication method or the morphology of the scaffolds.

An increase in cell metabolic activity and proliferation in the presence of low concentrations of 2dDr in solution has been shown before for aortic endothelial cells [53]. Furthermore, its potential as an angiogenic agent has been recently explored using in vitro and in vivo models [37,38,44,54]. Our results showed that this behavior can also be observed for HDF, further proving the versatility of 2dDr for tissue engineering applications. E2 has also been studied for its effects in wound healing, HDF migration, HDF proliferation, collagen synthesis, and production of TFG-β1 [55,56,57]. The mild change in metabolic activity and proliferation of HDF cultured with solubilized E2 indicates that nM concentrations are required to induce a higher cell activity [39].

The culture of dermal cells with AV has proved to increase the production of TGF and bFGF, both key factors in the wound healing process that mediate collagen deposition, HDF proliferation, and angiogenesis [58]. Our results showed that AV concentrations lower than 5 mg/mL had a significant increase in both metabolic activity and proliferation for HDF, with concentrations above 5 mg/mL having a negative effect on cell behavior. The effects of AV improving cell response at low concentrations had been reported before by Hormozi et al. and Zandit et al. in which µg/mL concentrations of AV showed to increase cell metabolic activity of embryonic mouse fibroblasts and ovine fibroblasts respectively [58,59]. Additionally, AV has been found to stimulate the expression of vascular endothelial growth factor and keratinocyte growth factor-1, both key components of the wound healing process [60].

Based on our in vitro data on tissue culture plastic, we have demonstrated that the HDK + HDF co-culture is less sensitive to the concentrations tested as shown by our preliminary experiments with HDK + HDF seeded on RES (Supplementary Materials Figure S1). Therefore, we used the HDF culture to study the changes in cell viability when seeded on RES loaded with bioactive compounds. Our cell activity data indicated that similar concentrations loaded into electrospun scaffolds have the potential to stimulate a similar increase in metabolic activity. However, there was no increase in HDF cell viability among the RES scaffolds loaded with bioactive compounds when compared with the PCL control. In contrast with the increase in metabolic activity of 3T3-L1 fibroblasts reported by Unnithan et al. [61], the high concentration of E2 (approximately 1 mg per scaffold) hindered the metabolic activity when compared with the AV and 2dDr scaffolds that recovered at day 6.

Although the potential of delivering two bioactive compounds within the same construct is an attractive approach that we would continue to explore in the future, the present TCES fabrication method did not allow the inclusion of more than one compound within the electrospun fibers. Our previous work showed that HDF seeded on TCES had no significant changes in metabolic activity. In contrast, HDK + HDF are susceptible to microtographical cues present on the scaffolds and thus a better model to study the effects of the TCES loaded with bioactive compounds [21].

Interestingly, HDK + HDF showed the highest metabolic avidity among the bioactive compounds when seeded on TCES loaded with E2. Whereas the presence of 2dDr and AV showed a significant decrease in metabolic activity. Although the presence of E2 has proved to be beneficial for keratinocyte cell survival and proliferation [62,63,64], the PCL control showed higher metabolic activity than the 10% E2 group, suggesting that a lower dosage might be needed to further improve cell behavior. A similar hypothesis of a high dosage can explain the decrease in HDK metabolic activity when exposed to 2dDr, however, the effects of 2dDr on HDK proliferation, migration, and cell metabolic activity that had not been reported before and are yet to be fully characterized.

Moreover, µM concentrations of some AV components have been identified as inhibitors of HDK proliferation [65], while other studies have shown that AV accelerates migration and proliferation of HDK [66,67,68], highlighting the synergistic interaction of the AV compounds [69]. Furthermore, AV has been used to treat psoriasis; a dermal condition characterized by the elongation of the rete ridges. Our work here shows the potential alternative to reintroduce the rete ridges dermal microenvironment while delivering a drug that inhibits the abnormal proliferation of HDK [70].

Lightsheet images showed that the TCES control and E2 groups preserved the microstructure even after 6 days in cell culture. Furthermore, the expression of Iβ1 was localized at the tip or base of the microfeatures for the TCES control and E2 groups, suggesting that HDK behavior was affected by the introduction of the microenvironments and that the bioactive compounds influence the expression of relevant biomolecules. In comparison, there is no distinct expression pattern of Iβ1 for cells seeded on RES regardless of the bioactive compound loaded into the scaffold. Although differences in cell proliferation can be observed among RES and TCES groups, a future immunohistochemical analysis would be needed to quantify the expression of markers as a function of location within the scaffold.

Our data from the CAM assay showed similar results to those reported before regarding the angiogenic potential of 2dDr, E2, and AV [37,38,39,71]. Although there was no statistical significance among the bioactive compounds, all of them showed a higher vascular density around the scaffold compared to the pure PCL control. In the case of 2dDr, Dicki et al. has shown that concentrations as high as 50% wt. can induce a mild angiogenic response. Our data further demonstrated the dosage-dependent nature of 2dDr, as our lower 10% wt. concentration induced a higher angiogenic response than the 50% wt. concentration [38]. In contrast, lower 5% wt. concentrations of E2 have proved to be enough to induce blood vessel formation on CAM with concentrations above that threshold generating a lower angiogenic response [38,39].

Interestingly, to the best of our knowledge, this is the first time that AV is loaded into electrospun scaffolds and studied under the CAM assay, as other studies have focused on the effects of individual compounds or the entire AV gel [71]. Our data showed the potential of these AV-loaded scaffolds to induce blood vessel formation using a reliable delivery method. However, characterizing the release kinetics of the RES as well as studying any differences compared to the TCES is key to fully understand the angiogenic capabilities of our scaffolds. In this research, we have demonstrated the potential of using our angiogenic microfabricated scaffolds as tools for the development of future complex skin tissue engineering devices.

## 5. Conclusions

In this study we have explored how to enhance the bioactivity of PCL electrospun scaffolds via the addition of key angiogenic compounds for then increase the complexity of these scaffolds via the inclusion of rete ridge-like structures that emulate to an extent the morphology of the stem cell niche in the skin. Furthermore, the dose-dependent response of the compounds was confirmed using HDF and HDF + HDK in vitro cultures.

Our methodology shows that it is possible to fabricate reproducible TCES with well-defined fibrous microenvironments that are also biofunctionalized with E2 as an angiogenic agent. Although fibers loaded with 2dDr and AV produced TCES with less-defined microenvironments, these membranes showed no decrease in cell proliferation when observed under the Lightsheet microscope. Our research demonstrates that the manufactured RES loaded with bioactive compounds can affect cell proliferation and induce blood vessel formation on the CAM assay making them a potential tool to be used in the future fabrication of complex skin tissue engineering devices.

## Figures and Tables

**Table 1:** PCL polymer solutions loaded with bioactive compounds.

Code	Solvent System	Bioactive Compound	Bioactive Compound % wt.
S1	DCM:DMF 3:1	N/A	N/A
S2		2dDr	8%
S3			10%
S4			15%
S5		E2	8%
S6			10%
S7	DCM:DMF + DMSO (3:1) + 500 µL	AV	5%
S8			10%
S9		AV + 2dDr	10% + 8%

**Table 2:** Electrospinning process parameters for solutions loaded with bioactive compounds.

Code	Voltage	Flow Rate	Code	Voltage	Flow Rate
S1	19–22 kV	1–4 mL/h	S6	19–21 kV	1–4 mL/h
S2	20–22 kV		S7	19–21 kV	
S3			S8	21–23 kV	
S4			S9	18–19 kV	3–4 mL/h
S5	19–21 kV				

**Table 3:** Polymer solutions and electrospinning process parameters used to fabricate TCES.

Code	Bioactive Compound and % wt	Voltage	Flow Rate
S1	N/A	19 kV	3–5 mL/h
S2	2dDr 8%	20–21 kV	
S6	E2 10%	19 kV	
S8	AV 10%	21–22 kV	

## Data Availability

Not applicable.

## Supplementary Materials

The following are available online at https://www.mdpi.com/article/10.3390/bioengineering8080105/s1. Supplementary Table S1: Supplements used to prepare DMEM and Green’s cell culture media. Reagents were purchased from Sigma (UK) unless specified. Supplementary Figure S1: Cell metabolic activity of HDF + HDK seeded on PCL RES loaded with 2dDr. ns = no significance. n = 3.

## References

[1] Blais M., Parenteau-Bareil R., Cadau S., Berthod F. (2013). Concise Review: Tissue-Engineered Skin and Nerve Regeneration in Burn Treatment. Stem Cells Transl. Med..

[2] Magin C.M., Neale D.B., Drinker M.C., Willenberg B.J., Reddy S.T., La Perle K.M., Schultz G.S., Brennan A.B. (2016). Evaluation of a bilayered, micropatterned hydrogel dressing for full-thickness wound healing. Exp. Biol. Med..

[3] Hughes O.B., Rakosi A., MacQuhae F., Herskovitz I., Fox J.D., Kirsner R.S. (2016). A Review of Cellular and Acellular Matrix Products: Indications, techniques, and outcomes. Plast. Reconstr. Surg..

[4] Wang Y., Beekman J., Hew J., Jackson S., Issler-Fisher A.C., Parungao R., Lajevardi S.S., Li Z., Maitz P.K. (2018). Burn injury: Challenges and advances in burn wound healing, infection, pain and scarring. Adv. Drug Deliv. Rev..

[5] Dias J.R., Granja P.L., Bartolo P.J. (2016). Advances in electrospun skin substitutes. Prog. Mater. Sci..

[6] Zhou H., You C., Wang X., Jin R., Wu P., Li Q., Han C. (2017). The progress and challenges for dermal regeneration in tissue engineering. J. Biomed. Mater. Res. Part A.

[7] MacNeil S. (2008). Biomaterials for tissue engineering of skin. Mater. Today.

[8] MacNeil S. (2007). Progress and opportunities for tissue-engineered skin. Nat. Cell Biol..

[9] Rouwkema J., Rivron N.C., van Blitterswijk C. (2008). Vascularization in tissue engineering. Trends Biotechnol..

[10] Ishak S.A., Djuansjah J.R.P., Kadir M.R.A., Sukmana I. (2014). Angiogenesis in tissue engineering: From concept to the vascularization of scaffold construct. IOP Conf. Ser. Mater. Sci. Eng..

[11] Frueh F.S., Menger M.D., Lindenblatt N., Giovanoli P., Laschke M.W. (2016). Current and emerging vascularization strategies in skin tissue engineering. Crit. Rev. Biotechnol..

[12] Blackwood K.A., McKean R., Canton I., Freeman C.O., Franklin K.L., Cole D., Brook I., Farthing P., Rimmer S., Haycock J. (2008). Development of biodegradable electrospun scaffolds for dermal replacement. Biomaterials.

[13] Abrigo M., McArthur S.L., Kingshott P. (2014). Electrospun Nanofibers as Dressings for Chronic Wound Care: Advances, Challenges, and Future Prospects. Macromol. Biosci..

[14] Pu J., Yuan F., Li S., Komvopoulos K. (2015). Electrospun bilayer fibrous scaffolds for enhanced cell infiltration and vascularization in vivo. Acta Biomater..

[15] Thakur R.A., Florek C.A., Kohn J., Michniak B.B. (2008). Electrospun nanofibrous polymeric scaffold with targeted drug release profiles for potential application as wound dressing. Int. J. Pharm..

[16] Luraghi A., Peri F., Moroni L. (2021). Electrospinning for drug delivery applications: A review. J. Control. Release.

[17] Xue J., Wu T., Dai Y., Xia Y. (2019). Electrospinning and Electrospun Nanofibers: Methods, Materials, and Applications. Chem. Rev..

[18] Ortega I., Ryan A.J., Deshpande P., MacNeil S., Claeyssens F. (2013). Combined microfabrication and electrospinning to produce 3-D architectures for corneal repair. Acta Biomater..

[19] Ramos-Rodriguez D.H., MacNeil S., Claeyssens F., Asencio I.O. (2021). The Use of Microfabrication Techniques for the Design and Manufacture of Artificial Stem Cell Microenvironments for Tissue Regeneration. Bioengineering.

[20] Paterson T.E., Beal S.N., Santocildes-Romero M.E., Sidambe A.T., Hatton P.V., Asencio I.O. (2017). Selective laser melting-enabled electrospinning: Introducing complexity within electrospun membranes. Proc. Inst. Mech. Eng. Part H J. Eng. Med..

[21] Ramos-Rodriguez D.H., MacNeil S., Claeyssens F., Ortega Asencio I. (2021). Fabrication of Topographically Controlled Electrospun Scaffolds to Mimic the Stem Cell Microenvironment in the Dermal-Epidermal Junction. ACS Biomater. Sci. Eng..

[22] Ortega Ascensio I., Mittar S., Sherborne C., Raza A., Claeyssens F., MacNeil S. (2018). A methodology for the production of microfabricated electrospun membranes for the creation of new skin regeneration models. J. Tissue Eng..

[23] Shukla A.K., Dey N., Nandi P., Ranjan M. (2015). Acellular Dermis as a Dermal Matrix of Tissue Engineered Skin Substitute for Burns Treatment. Ann. Public Health Res..

[24] Clement A.L., Pins G.D., Agren M. (2016). Engineering the tissue-wound interface: Harnessing topography to direct wound healing. Wound Healing Biomaterials-Volume 1: Therapies and Regeneration.

[25] Clement A.L., Moutinho T.J., Pins G.D. (2013). Micropatterned dermal-epidermal regeneration matrices create functional niches that enhance epidermal morphogenesis. Acta Biomater..

[26] Lutolf M.P., Blau H.M. (2009). Artificial Stem Cell Niches. Adv. Mater..

[27] Rezza A., Sennett R., Rendl M. (2014). Adult stem cell niches: Cellular and molecular components. Curr. Top. Dev. Biol..

[28] Boyle M., Wong C., Rocha M., Jones D.L. (2007). Decline in Self-Renewal Factors Contributes to Aging of the Stem Cell Niche in the Drosophila Testis. Cell Stem Cell.

[29] Hsu H.-J., Drummond-Barbosa D. (2011). Insulin signals control the competence of the Drosophila female germline stem cell niche to respond to Notch ligands. Dev. Biol..

[30] Smith-Berdan S., Nguyen A., Hassanein D., Zimmer M., Ugarte F., Ciriza J., Li D., García-Ojeda M.E., Hinck L., Forsberg E.C. (2011). Robo4 cooperates with Cxcr4 to specify hematopoietic stem cell localization to bone marrow niches. Cell Stem Cell.

[31] Avigdor A., Goichberg P., Shivtiel S., Dar A., Peled A., Samira S., Kollet O., Hershkoviz R., Alon R., Hardan I. (2004). CD44 and hyaluronic acid cooperate with SDF-1 in the traf cking of human CD34+ stem/progenitor cells to bone marrow. Blood.

[32] Ellis S.J., Tanentzapf G. (2009). Integrin-mediated adhesion and stem-cell-niche interactions. Cell Tissue Res..

[33] Banks J.M., Harley B.A.C., Bailey R.C. (2015). Tunable, Photoreactive Hydrogel System to Probe Synergies between Mechanical and Biomolecular Cues on Adipose-Derived Mesenchymal Stem Cell Differentiation. ACS Biomater. Sci. Eng..

[34] Alsberg E., A Von Recum H., Mahoney M.J. (2006). Environmental cues to guide stem cell fate decision for tissue engineering applications. Expert Opin. Biol. Ther..

[35] Zhang Y., Gordon A., Qian W., Chen W. (2015). Engineering Nanoscale Stem Cell Niche: Direct Stem Cell Behavior at Cell-Matrix Interface. Adv. Health Mater..

[36] McNamara L.E., McMurray R.J., Biggs M.J.P., Kantawong F., Oreffo R., Dalby M.J. (2010). Nanotopographical Control of Stem Cell Differentiation. J. Tissue Eng..

[37] Andleeb A., Dikici S., Waris T.S., Bashir M.M., Akhter S., Chaudhry A.A., MacNeil S., Yar M. (2020). Developing affordable and accessible pro-angiogenic wound dressings; incorporation of 2 deoxy D-ribose (2dDR) into cotton fibres and wax-coated cotton fibres. J. Tissue Eng. Regen. Med..

[38] Dikici S., Mangir N., Claeyssens F., Yar M., MacNeil S. (2019). Exploration of 2-deoxy-D-ribose and 17β-Estradiol as alternatives to exogenous VEGF to promote angiogenesis in tissue-engineered constructs. Regen. Med..

[39] Shafaat S., Mangir N., Regureos S.R., Chapple C.R., MacNeil S. (2018). Demonstration of improved tissue integration and angiogenesis with an elastic, estradiol releasing polyurethane material designed for use in pelvic floor repair. Neurourol. Urodyn..

[40] Carter P., Rahman S.M., Bhattarai N. (2016). Facile fabrication of aloe vera containing PCL nanofibers for barrier membrane application. J. Biomater. Sci. Polym. Ed..

[41] Sánchez-Machado D.I., López-Cervantes J., Sendon R., Silva A.S. (2017). Aloe vera: Ancient knowledge with new frontiers. Trends Food Sci. Technol..

[42] A Schneider C., Rasband W.S., Eliceiri K.W. (2012). NIH Image to ImageJ: 25 years of image analysis HHS public access. Nat. Methods.

[43] Ghosh M.M., Boyce S., Freedlander E., Mac Neil S., Layton C. (1997). A Comparison of Methodologies for the Preparation of Human Epidermal-Dermal Composites. Ann. Plast. Surg..

[44] Mangir N., Dikici S., Claeyssens F., MacNeil S. (2019). Using ex Ovo Chick Chorioallantoic Membrane (CAM) Assay to Evaluate the Biocompatibility and Angiogenic Response to Biomaterials. ACS Biomater. Sci. Eng..

[45] Elfarnawany M.H. (2015). Signal Processing Methods for Quantitative Power Doppler Microvascular Angiography. Ph.D. Thesis.

[46] Pillay V., Dott C., Choonara Y., Tyagi C., Tomar L., Kumar P., du Toit L., Ndesendo V.M.K. (2013). A Review of the Effect of Processing Variables on the Fabrication of Electrospun Nanofibers for Drug Delivery Applications. J. Nanomater..

[47] Repanas A., Andriopoulou S., Glasmacher B. (2016). The significance of electrospinning as a method to create fibrous scaffolds for biomedical engineering and drug delivery applications. J. Drug Deliv. Sci. Technol..

[48] Sill T.J., von Recum H.A. (2008). Electrospinning: Applications in drug delivery and tissue engineering. Biomaterials.

[49] Bravo J.M.C., Gómez L.J.V., Medina A.S. (2016). Electrospinning for drug delivery systems: Drug incorporation techniques. Electrospinning—Material, Techniques, and Biomedical Applications.

[50] Maan A.A., Nazir A., Khan M.K.I., Ahmad T., Zia R., Murid M., Abrar M. (2018). The therapeutic properties and applications of Aloe vera: A review. J. Herb. Med..

[51] Uslu S., Keskin A., Gül T., Karabulut C., Aksu M.L. (2010). Preparation and Properties of Electrospun Poly (vinyl alcohol) Blended Hybrid Polymer with Aloe vera and HPMC as Wound Dressing. Hacet. J. Biol. Chem..

[52] Kim C.H., Khil M.S., Kim H.Y., Lee H.U., Jahng K.Y. (2006). An improved hydrophilicity via electrospinning for enhanced cell attachment and proliferation. J. Biomed. Mater. Res. Part B Appl. Biomater..

[53] Dikici S., Bullock A., Yar M., Claeyssens F., MacNeil S. (2020). 2-deoxy-d-ribose (2dDR) upregulates vascular endothelial growth factor (VEGF) and stimulates angiogenesis. Microvasc. Res..

[54] Dikici S., Dikici B.A., Bhaloo S.I., Balcells M., Edelman E., MacNeil S., Reilly G.C., Sherborne C., Claeyssens F. (2020). Assessment of the Angiogenic Potential of 2-Deoxy-D-Ribose Using a Novel in vitro 3D Dynamic Model in Comparison with Established in vitro Assays. Front. Bioeng. Biotechnol..

[55] Stevenson S., Nelson L.D., Sharpe D.T., Thornton M.J. (2008). 17β-Estradiol regulates the secretion of TGF-β by cultured human dermal fibroblasts. J. Biomater. Sci. Polym. Ed..

[56] Makrantonaki E., Vogel K., Fimmel S., Oeff M., Seltmann H., Zouboulis C.C. (2008). Interplay of IGF-I and 17β-estradiol at age-specific levels in human sebocytes and fibroblasts in vitro. Exp. Gerontol..

[57] Surazynski A., Jarzabek K., Haczynski J., Laudanski P., Palka J., Wolczynski S. (2003). Differential effects of estradiol and raloxifene on collagen biosynthesis in cultured human skin fibroblasts. Int. J. Mol. Med..

[58] Hormozi M., Assaei R., Boroujeni M.B. (2017). The effect of aloe vera on the expression of wound healing factors (TGFβ1 and bFGF) in mouse embryonic fibroblast cell: In vitro study. Biomed. Pharmacother..

[59] Zandi M., Masoumian M., Shariatinia A., Sanjabi M.R. (2016). Optimal Concentrations and Synergistic Effects of Some Herbal Extracts on Viability of Dermal Fibroblasts. Gene Cell Tissue.

[60] Jettanacheawchankit S., Sasithanasate S., Sangvanich P., Banlunara W., Thunyakitpisal P. (2009). Acemannan stimulates gingival fibroblast proliferation; expressions of keratinocyte growth factor-1, vascular endothelial growth factor, and type I collagen; and wound healing. J. Pharmacol. Sci..

[61] Unnithan A.R., Sasikala A.R.K., Murugesan P., Gurusamy M., Wu D., Park C.H., Kim C.S. (2015). Electrospun polyurethane-dextran nanofiber mats loaded with Estradiol for post-menopausal wound dressing. Int. J. Biol. Macromol..

[62] Urano R., Sakabe K., Seiki K., Ohkido M. (1995). Female sex hormone stimulates cultured human keratinocyte proliferation and its RNA- and protein-synthetic activities. J. Dermatol. Sci..

[63] Kanda N., Watanabe S. (2003). 17β-Estradiol Inhibits Oxidative Stress-Induced Apoptosis in Keratinocytes by Promoting Bcl-2 Expression. J. Investig. Dermatol..

[64] Verdier-Sevrain S., Yaar M., Cantatore J., Traish A., Gilchrest B.A. (2004). Estradiol induces proliferation of keratinocytes via receptor-mediated mechanisms. FASEB J..

[65] Popadic D., Savic E., Ramic Z., Djordjevic V., Trajkovic V., Medenica L., Popadic S. (2012). Aloe-emodin inhibits proliferation of adult human keratinocytes in vitro. J. Cosmet. Sci..

[66] Moriyama M., Moriyama H., Uda J., Kubo H., Nakajima Y., Goto A., Akaki J., Yoshida I., Matsuoka N., Hayakawa T. (2016). Beneficial Effects of the Genus Aloe on Wound Healing, Cell Proliferation, and Differentiation of Epidermal Keratinocytes. PLoS ONE.

[67] Choi S.-W., Son B.-W., Son Y.-S., Park Y.-I., Lee S.-K., Chung M.-H. (2001). The wound-healing effect of a glycoprotein fraction isolated from aloe vera. Br. J. Dermatol..

[68] Takahashi M., Kitamoto D., Asikin Y., Takara K., Wada K. (2009). Liposomes encapsulating Aloe vera leaf gel extract significantly enhance proliferation and collagen synthesis in human skin cell lines. J. Oleo Sci..

[69] Hamman J.H. (2008). Composition and Applications of Aloe vera Leaf Gel. Molecules.

[70] Leng H., Pu L., Xu L., Shi X., Ji J., Chen K. (2018). Effects of aloe polysaccharide, a polysaccharide extracted from Aloe vera, on TNF-α-induced HaCaT cell proliferation and the underlying mechanism in psoriasis. Mol. Med. Rep..

[71] Moon E.-J., Lee Y.M., Lee O.-H., Lee M.-J., Lee S.-K., Chung M.-H., Park Y.-I., Sung C.-K., Choi J.-S., Kim K.-W. (1999). A ncovel angiogenic factor derived from Aloe vera gel: β-sitosterol, a plant sterol. Angiogenesis.

