# Supplementary material for: Delivery of Bioactive Compounds to Improve Skin Cell Responses on Microfabricated Electrospun Microenvironments
Source: Bioengineering (Basel). 2021 Jul 27;8(8):105. doi: 10.3390/bioengineering8080105 (PMC8389211; doi:10.3390/bioengineering8080105)
Supplement: Supplementary file 1 [file bioengineering-08-00105-s001.zip › Supplementary Table S1.pdf]

Table S1. Supplements used to prepare DMEM and Green's cell culture media. Reagents were purchased from Sigma (UK) unless specified.

Supplement	DMEM (6546)	Green's media
Foetal calf serum (Biowest Biosera)	10% v/v	
Penicillin- streptomycin	100 IU/mL - 100 mg/mL	
Amphotericin B	0.625 μg/mL	
Ham's F12 (N4888)	N/A	3:1 v/v DMEM AQ : Ham's F12
DMEM AQ media (D0819)		
L-glutamine	2 mM	N/A
Bovine insulin	N/A	5 μg/mL
Adenine		6.25 μg/mL
T/T		10 ng/mg
Hydrocortisone		0.4 μg/mL
EGF		10 ng/mL
Cholera toxin		8.5 ng/mL
