# Supplementary figures and images for: Delivery of Bioactive Compounds to Improve Skin Cell Responses on Microfabricated Electrospun Microenvironments
Source: Bioengineering (Basel). 2021 Jul 27;8(8):105. doi: 10.3390/bioengineering8080105 (PMC8389211; doi:10.3390/bioengineering8080105)

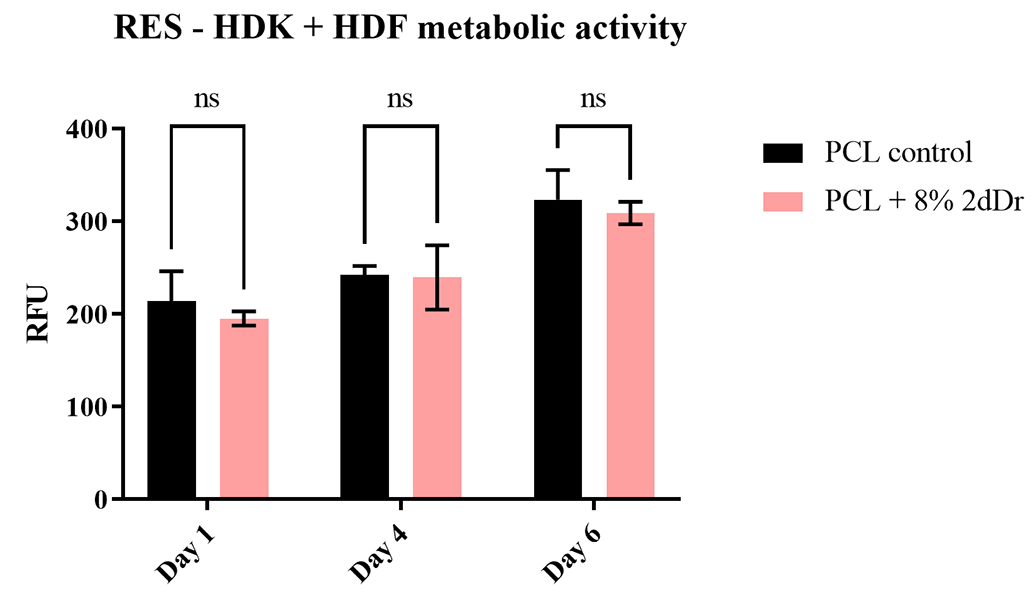

Supplement: Supplementary file 1 [file bioengineering-08-00105-s001.zip › Supplementary Figure S1.tif]
